# An iron-chelating sulfonamide identified from *Drosophila*-based screening for antipathogenic discovery

**DOI:** 10.1080/21505594.2022.2069325

**Published:** 2022-05-06

**Authors:** Yeon-Ji Yoo, In-Young Chung, Shivakumar S. Jalde, Hyun-Kyung Choi, You-Hee Cho

**Affiliations:** a Department of Pharmacy, College of Pharmacy and Institute of Pharmaceutical Sciences, CHA University, Seongnam, Korea; b Department of Chemistry, Sogang University, Seoul, Korea

**Keywords:** Iron, chelator, catechol, sulfonamide, antipathogenic, virulence, *Drosophila*, *Pseudomonas aeruginosa*

## Abstract

We exploited bacterial infection assays using the fruit fly *Drosophila melanogaster* to identify anti-infective compounds that abrogate the pathological consequences in the infected hosts. Here, we demonstrated that a pyridine-3-*N*-sulfonylpiperidine derivative (**4a**) protects *Drosophila* from the acute infections caused by bacterial pathogens including *Pseudomonas aeruginosa*. **4a** did not inhibit the growth of *P. aeruginosa* in vitro, but inhibited the production of secreted toxins such as pyocyanin and hydrogen cyanide, while enhancing the production of pyoverdine and pyochelin, indicative of iron deprivation. Based on its catechol moiety, **4a** displayed iron-chelating activity in vitro toward both iron (II) and iron (III), more efficiently than the approved iron-chelating drugs such as deferoxamine and deferiprone, concomitant with more potent antibacterial efficacy in *Drosophila* infections and unique transcriptome profile. Taken together, these results delineate a *Drosophila*–based strategy to screen for antipathogenic compounds, which interfere with iron uptake crucial for bacterial virulence and survival in host tissues.

## GRAPHICAL ABSTRACT

**Figure:**
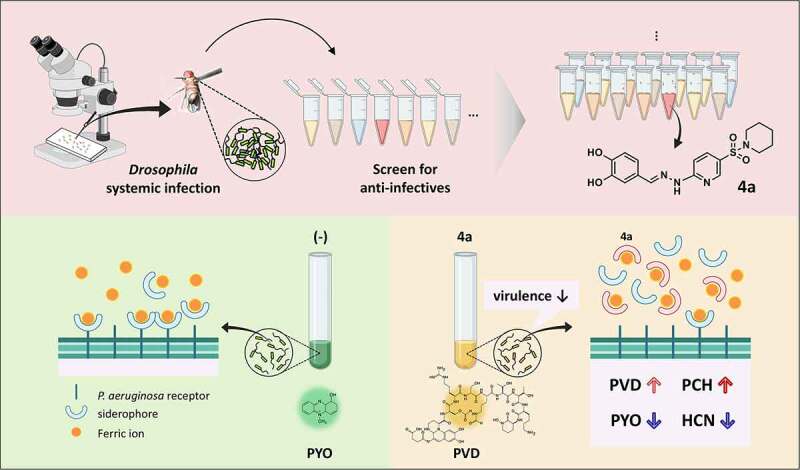

## Introduction

Multidrug-resistant (MDR) bacterial pathogens have spread worldwide at an alarming rate and causing many types of diseases [1,2], which is one of the major public health challenges especially in this era of pandemic infections. ESKAPE pathogens (*Enterococcus faecium*, *Staphylococcus aureus*, *Klebsiella pneumoniae*, *Acinetobacter baumannii*, *Pseudomonas aeruginosa,* and *Enterobacter* species) are of particular concern, in that they are associated with MDR nosocomial infections, escaping the currently available antibiotic regimen [3]. In that the resistance emergence especially in the ESKAPE pathogens is inevitable due to the selective mutation and/or horizontal acquisition for resistance, new-paradigm approaches rather than the traditional ones have received much attention for antibacterial discovery [4]. One increasingly compelling approach is to attenuate bacterial virulence without killing or inhibiting the growth of bacteria, as it is thought to confer limited selective pressure for the resistance emergence [5].

Bacterial pathogens exploit an arsenal of virulence factors to cause diseases in the human host. These factors are any bacterial determinants involved in the various stages of bacterial pathogenesis, which include adhesion, invasion, colonization, and toxin production [5,6]. The factors required to resist or tolerate the host defenses are also important for pathogenesis, and thus the target for antipathogenic strategy. Among those virulence factors, the quorum-sensing systems are noteworthy, in that they are responsible for regulation of multiple virulence factors, involving the secreted chemicals called quormones, which could be targeted even without entry into the target pathogens [7,8]. Recently, we have identified a chemical hit to compromise the peroxide-sensor, OxyR through structure-based virtual screening [9] and developed an antipathogenic peptide that is derived from a phage protein targeting the bacterial motility and acute virulence [10,11].

In contrast to the target-based approaches, researchers have been developing the phenotype-based approaches using small-scale live non-mammalian model host infections to screen for new classes of anti-infective chemicals such as antipathogenic ones [12,13]. The main readout of the live animal infection screens is the survival of the infected hosts rather than the death of the infecting bacteria. Moreover, using live animals offers an in-assay counter-screen against the compounds with toxicity and poor pharmacokinetics, which would be another advantage of these live animal infection screening platforms [14,15], as developed using the nematode *Caenorhabditis elegans* and the zebrafish *Danio rerio* [16,17].

In this study, we exploited the fruit fly *Drosophila melanogaster*-based screens using a set of in-house chemicals with a hope to identify antipathogenic hits against either of two ESKAPE pathogens, *P. aeruginosa* and methicillin-resistant *S. aureus*. As a result, a pyridine-3-sulfonamide derivative (**4a**) was identified as a hit that possesses antipathogenic activity against *P. aeruginosa*, affecting not the growth but the toxin production of this bacterium. **4a** contains a catechol moiety which provides iron-chelating activity to reduce the iron availability for the bacterial virulence. Comparison with other chelating drugs and its nonfunctional sulfonamide analogs revealed the unique feature of **4a** as an antipathogenic iron-chelator.

## Materials and methods

### Chemical compounds

All of the commercial chemicals and solvents are of reagent grade and were used without further purification. Iron chelating drugs, deferoxamine (DFA) and deferiprone (DFP), were purchased (Sigma). In-house chemical compounds were synthesized and purified by column chromatograpy. Their structures were verified throwth ^1^H and ^13^C NMR spectra and mass spectrometry. Both UV and IR equipments were provided by Advanced Bio-Interface Core Research Facility at Sogang University, Seoul, South Korea.

### Bacterial strains and culture conditions

The bacterial strains used in this study are *Pseudomonas aeruginosa* PA14, *Vibrio cholerae* N16961, *Staphylococcus aureus* SA3 [18]. Cells were grown at 37°C using Luria-Bertani (LB) (0.5% yeast extract, 1% tryptone, and 1% NaCl) broth, Müller-Hinton (MH) broth, and M9-citrate minimal medium (0.05% NaCl, 0.1% NH_4_Cl, 1.2% Na_2_HPO_4_, 0.3% KH_2_PO_4_, 2 mM MgSO_4_, 0.1 mM CaCl_2_, and 0.4% citrate) or on 2% LB agar plates. In general, overnight-grown cultures were used as inoculum (1.6 × 10^7^ cfu/ml) into fresh LB and grown at 37°C in a shaking incubator until the logarithmic phase (i.e. OD_600_ of 0.7), and then used for the experiments.

For growth curve determination, *P. aeruginosa* PA14 cells were used as inoculum (2.0 × 10^8^ cfu/ml) into LB broth with or without 500 μM **4a** and the cultures were incubated in the microplate reader (EPOCH2, USA) at 37°C for 16 h. OD_600_ was measured at every 20 min during the incubation The viability was also assessed by spotting ten-fold serial dilutions (3 μl) of the 16-h cultures onto an LB agar.

### *Drosophila* experiments

*Drosophila melanogaster* (Oregon R) was grown and maintained as described previously [19], using corn meal-dextrose medium [0.93% agar, 6.24% dry yeast, 4.08% corn meal, 8.62% dextrose, 0.1% methyl paraben, and 0.45% (v/v) propionic acid]. For *Drosophila*-based screening, small-scale systemic infection was performed as previously described [20] with slight modification. Briefly, 4- to 5-day-old flies were infected by pricking at the dorsal thorax with a 0.4 mm needle, which had been dipped into PBS-diluted bacterial suspension containing *P. aeruginosa* PA14 (10^7^ cfu/ml), *V. cholerae* N16961 (5 × 10^7^ cfu/ml) or *S. aureus* SA3 (10^7^ cfu/ml) grown to the OD_600_ of 3.0. Groups of six infected flies were transferred to the fly medium containing one (500 µM) out of 444 in-house chemicals and drugs from various sources and then monitored up to 50 h. The tubes with more than two survivors were selected as containing primary hits. For the secondary screening, antibacterial efficacy of the chemicals was assessed as described elsewhere [10]. The female flies were pre-incubated in a new medium overlaid with a chemical (500 µM) and then infected as above. Survival rates of the infected flies were monitored for up to 50 h post-infection. Flies that died within 12 h for PA14 and SA3, 6 h for N16961 were excluded in mortality determination. Each result shown is a pool of data from three independent biological replicates.

### Measurement of minimal inhibitory concentrations

Minimal inhibitory concentrations (MICs) for **4a**, gentamicin, and carbenicillin were determined in MH broth by broth microdilution according to NCCLS guidelines, as described elsewhere [11]. The medium, with a 2-fold serial dilution of each compound (**4a**, 3.9 µM to 1 mM; carbenicillin, 9.8 µM to 2.5 mM; gentamicin, 7.8 µM to 2 mM) in MH broth, was subjected to inoculation with PA14 (5 × 10^5^ CFU/ml) that had been grown at 37°C to OD_600_ of 1.0 and then incubated at 37°C. The MIC values were recorded as the lowest concentration of the chemical at which no signs of growth were observed, based on the OD_600_ value of less than 0.05 after 18 h of incubation. The MIC values were confirmed by three independent experiments.

### Measurement of pigment production

Production of pyocyanin (PYO) and pyoverdine (PVD) was colorimetrically enumerated using late stationary phase or overnight-grown cultures of PA14 as described elsewhere [21,22]. For PYO measurement, chloroform (1 eq) was added to the cell-free supernatant to extract PYO into the chloroform layer. The chloroform layer was mixed with 0.2 N HCl (1 eq), which was subjected to OD_510_ measurement. For PVD measurement, the cell-free supernatant was appropriately diluted in 0.1 M Tris-HCl (pH 8), which was subjected to OD_405_ measurement. The corresponding OD_600_ values were used to normalize the measured values [23].

### Iron repletion and chelation

M9-citrate media without trace element solution were used for iron repletion at the indicated concentrations of either FeSO_4_ or FeCl_3_. For iron chelation assay, serial 2-fold dilutions of FeSO_4_ or FeCl_3_ were prepared in TDW (from 0.6 μM to 10 mM) and mixed with 500 μM of iron chelators. The iron chelates were precipitated by centrifugation.

### RNA sequencing and transcriptome analysis

RNA was isolated from the logarithmic-growth phase cultures (OD_600_ of 0.7) of PA14, suing Trizol (Invitrogen). In addition, we treated eluted RNA with DNase I for 1 h at 37°C Ribosomal RNA was removed using Ribo-Zero Magnetic kit (epicenter, Inc., USA) from total RNA (5 μg). Library construction followed by the subsequent experimental steps for HiSeq2500 (illumina, Inc USA)-based high-throughput RNA-sequencing was carried out by a local company (ebiogen, Korea).

Two biologically independent experiments were performed. Bacterial-Seq reads were aligned using Bowtie2 software tool. Differentially expressed genes (DEGs) were determined based on counts from unique and multiple alignments using EdgeR [24]. Quantile normalization method was used for comparison between samples. Gene classification was based on DAVID (https://david.ncifcrf.gov).

### Statistics

GraphPad Prism version 8.0 (GraphPad Software, USA) was used for statistical analysis. Data sets for each analysis represent more than three independent replicates. Statistical significance between the groups is indicated, based on a *p* value of less than 0.001 (***, *p* < 0.001) by using Kaplan–Meier log-rank test or Student’s *t* test. Error bars represent the standard deviations. Gene expression changes were identified with statistical significance, based on the Mann–Whitney U test, with a cutoff *p* value of 0.05 [25].

## Results

### An antibacterial hit identified from Drosophila screens is not growth-inhibitory

**Figure 1.**
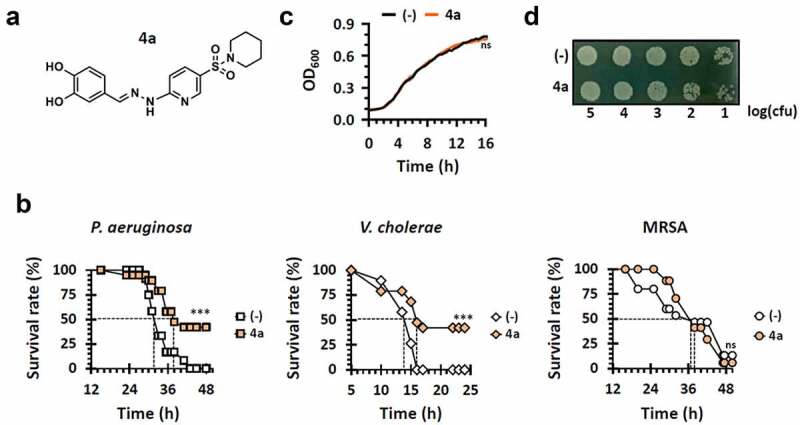
Identification of an antipathogenic hit (4a). (a). Chemical structure of **4a**, (*E*)-4-((2-(5-(piperidin-1-ylsulfonyl)pyridin-2-yl)hydrazono)methyl)benzene-1,2-diol. (b). Antibacterial efficacy of **4a** in *Drosophila* systemic infection model. The flies were systemically infected with *P. aeruginosa* PA14, *V. cholerae* N16961, or *S. aureus* SA3 (MRSA). The infected flies were fed with either nothing (empty) or 500 μM **4a** (filled) and their survival rates were determined over time. The dotted lines represent the time required to reach 50% mortality. The statistical significance based on a log-rank test is indicated (***, *p* < 0.001). (c). Growth of *P. aeruginosa* PA14 in the presence of **4a**. PA14 cells were grown in either LB broth (-) and LB broth containing 500 μM **4a**. OD_600_ values were measured every 20 min for 16 h. The presented values are averages from the three independent experiments with standard deviations. n.s. indicates non-significant based on a *p* value of over than 0.05 by using Mann–Whitney test. (d). Viability of the 16-h cultures from (c). Ten-fold serial dilutions from the 16-h PA14 cultures in either LB broth (-) or LB broth containing 500 μM **4a** were spotted onto an LB agar plate. The numbers indicate the log(cfu) values of the applied bacterial spots, which were calculated from the OD_600_ values.

We established a *Drosophila melanogaster*-based infection model to discover new anti-infective chemicals [15]. *V. cholerae* (N16961), *S. aureus* (SA3) and *P. aeruginosa* (PA14) were systemically infected by pricking at dorsal thorax of the flies, placed in a medium containing a chemical compound to screen for the compounds that enhanced the survival rate of the infected flies. The overall screening strategy is summarized in Figure S1. As a result, a hit (**4a**) (Figure 1a) was identified with antibacterial efficacy in the infections caused by N16961 and PA14, but not by SA3 (Figure 1b). **4a** is ((3,4-dihydroxybenzylidene)hydrazinyl)pyridine-3-*N*-sulfonylpiperidine, whose congeneric sulfonamides have been studied as anticancer compounds [26]. **4a** contains a catechol moiety that is presumably involved in iron chelation [27]. Notably, **4a** did not display antibacterial activity against PA14 in vitro (Figure 1c,d and S2), suggesting the antibacterial efficacy in vivo should be attributed to either antipathogenic or immunomodulatory properties of **4a**, which will be further discussed below.

#### 4a affects pigment production in *P.* *aeruginosa*

**Figure 2.**
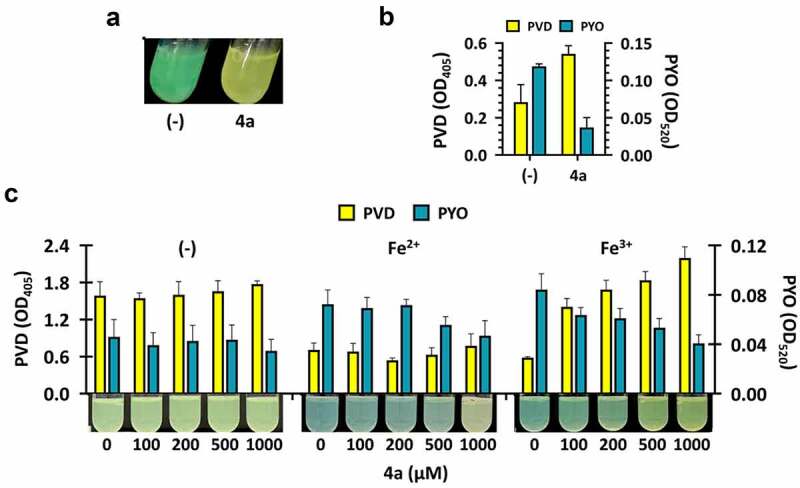
Pigment production by 4a. (a) and (b). Pyoverdine (PVD) and pyocyanin (PYO) production upon **4a** treatment in LB broth. PA14 cells were grown in LB broth with either nothing (-) or 500 μM **4a**. Pigment production of the 18-h cultures was assessed by visual inspection (a) or by quantification of PVD and PYO (b). The amounts of extracellular PVD (yellow, left y axis) and PYO (green, right y axis) were measured as OD_405_ and OD_520_ respectively. (c). PVD and PYO production upon **4a** treatment in M9-citrate broth under iron depletion (-) or repletion with either 5 μM FeSO_4_ (Fe^2+^) or FeCl_3_ (Fe^3+^). The amounts of extracellular PVD (yellow, left y axis) and PYO (green, right y axis) were measured as OD_405_ and OD_520_ respectively under **4a** treatment at the indicated concentrations. Pigment production of the cultures by visual inspection was shown at the bottom of the graphs.

In order to address the bioactivity of **4a**, we investigated the growth of PA14 in LB broth containing 200 µM **4a**. As shown in Figure 2a, the blue-green pigment pyocyanin (PYO) production was dramatically reduced. PYO is a redox-active compound considered as an important virulence factor in *P. aeruginosa* infections. PYO is generally reduced inside the cell and are oxidized outside the cell by iron (III) [28], which could facilitate iron solubilization into iron (II) and subsequently enhance the iron accessibility for the bacterial cells [29]. Moreover, the yellow-green fluorescence mostly attributed to pyoverdine (PVD) was apparently increased (Figure 2a). This observation was validated by quantification of both pigments (Figure 2b). Unlike LB medium that contains ~16 µM iron [30], PVD production was observed in the 16-hours culture of PA14 that had been grown in M9 minimal medium without iron supplementation as an iron-depleted medium (Figure S3). The amendment of either iron (II) or iron (III) at different concentrations to M9 gradually restored the production of PYO, indicating that iron supplementation at 10 µM would be exploited for further experiments.

We examined the effect of **4a** on the pigment production in 16-h culture of PA14 under iron-depleted and -repleted conditions using M9 (Figure 2c). Unlike that in the iron-depleted condition, the culture in the iron-repleted conditions with either iron (II) or iron (III) exhibited different PYO and PVD production profiles: both iron (II) and iron (III) reduced PYO production, whereas only iron (III) enhanced PVD production. This result substantiated the impact of **4a** on the secreted pigment production in *P. aeruginosa*, most likely by altering iron availability in the culture media.

#### 4a chelates both iron (II) and iron (III) in vitro

**Figure 3.**
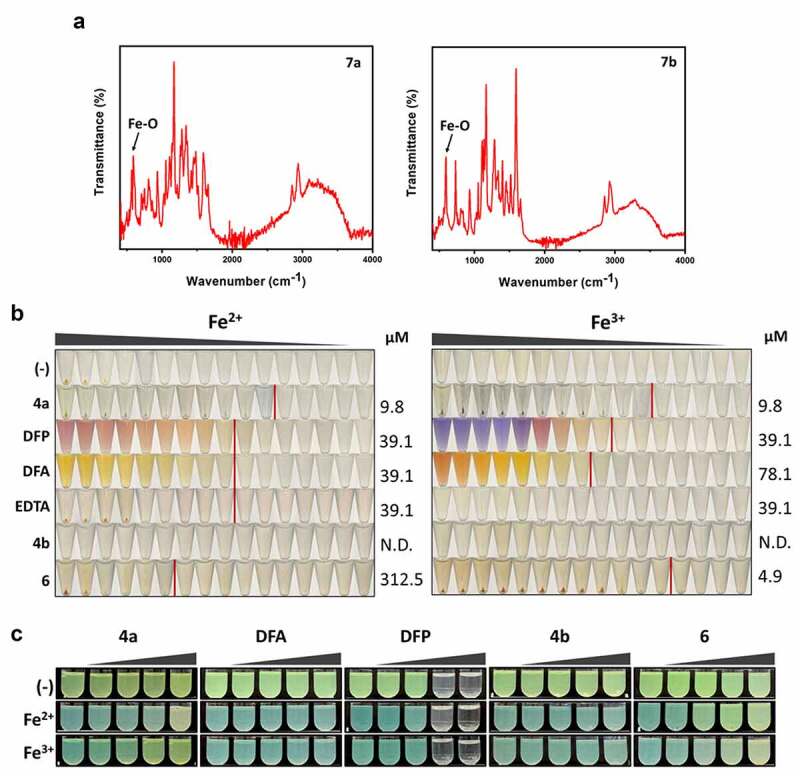
Iron chelation and pigment production by iron chelators. (a). IR spectra of the 4a chelated with irons. The peaks at 592 cm^−1^ (left) and 591 cm^−1^ (right) in the spectra could be allocated to the Fe-O bond formation in **7a** and **7b**, which has been generated as described in SI. (b). Iron chelation activities. Serial 2-fold dilutions of FeSO_4_ (Fe^2+^) or FeCl_3_ (Fe^3+^) were prepared in TDW (from 0.6 μM to 10 mM) and mixed with 500 μM of the designated compounds at the left: DFP, deferiprone, DFA, deferoxamine, EDTA, and ethylene-diamine-tetraacetic acid, as well as with the **4a** derivatives (**4b** and **6**). The minimum concentrations of chelation for the compounds are designated at the right, with the vertical red lines denoting the boundaries between the minimum concentrations of chelation and the maximal concentrations at which no chelation was observed. (c). Growth and pigment production upon the chemical treatment in M9-citrate broth under iron depletion (-) or repletion with either 5 μM FeSO_4_ (Fe^2+^) or FeCl_3_ (Fe^3+^). The growth and the pigment production of the PA14 cultures under chemical treatments at the increasing concentrations (0, 100, 200, 500, and 1,000 μM) was assessed by visual inspection.

To verify the iron chelation by **4a**, potential iron-chelated products were fabricated by using **4a** and both iron (III) (FeCl_3_^.^6 H_2_O) and iron (II) (FeSO_4_^.^7 H_2_O) to generate **7a** and **7b**, respectively, as described in Supplementary Methods. After fabrication, the **4a**-iron complexes were subjected to IR and UV-visible (UV-Vis) spectroscopy. As shown in Figure 3a the formation of iron chelation was verified by a strong stretching vibrational band at about 592 cm^−1^ (**7a**) and 591 cm^−1^ (**7b**) in the IR spectra, as the hall mark of the Fe-O bond formation [31]. Moreover, the absorption bands near 486 nm in the UV-Vis absorption spectra of **7a** and **7b** could be associated with a change in electronic distribution due to *d-d* transition (Figure S4) [31].

To further verify that **4a** does indeed chelate iron through its catechol moiety, we have synthesized two analogous chemicals (Figure S5): one is **4b** with two hydroxyl groups substituted by two methoxy groups, and the other is **6** with the catechol moiety with the hydroxamate moiety. It is well-known that hydroxamate is also capable of iron chelation exploited in some siderophores such as ferrichrome. In addition to these two **4a** analogs, other well-known chelating drugs and chemicals were as well used for comparison, which include deferiprone (DFP, a second-line drug in thalassemia syndromes), deferoxamine (DFA, a drug for acute iron intoxication and chronic iron overload due to transfusion-dependent anemias), and ethylene-diamine-tetraacetic acid (EDTA, a strong chelator for divalent cations). As shown in Figure 3b, **4a** displayed the most efficient chelating activity as assessed by insoluble precipitate formation. It chelates at 9.8 µM of both iron (II) and iron (III), whereas the chelating ability of DFP was 4-fold lower than that of **4a**. DFA displayed no less chelating activity that DFP toward iron (II), but 2-fold lower than that of DFP toward iron (III). As expected, EDTA chelated only iron (II), which was 4-fold less efficiently than **4a** did. It should be noted that **4b** did not display any chelating activity, suggesting that the catechol moiety is indeed required for the iron-chelation of **4a**. Moreover, **6** showed inferior activity toward iron (II), but superior activity toward iron (III) than **4a**, dissecting the chelating activities toward iron (II) and iron (III), based on the configurations that differ in **4a** and **6**.

Next, these compounds were tested for their bioactivity first regarding the pigment production under the iron-depleted and -repleted conditions as in Figure 2c (Figure 3c). It is quite interesting that unlike **4a**, the two iron-chelating drugs did not affect pigment production at all: DFP was rather growth-inhibitory at over 500 µM, whereas DFA had no effect on the growth either. The result for DFA was not surprising, since DFA may readily deliver iron to bacteria as a xenosiderophore [32]. It is evident that the iron-chelating activity in test tubes did not simply translate into the bioactivity in culture condition. It should be noted that **4b** had no effect on the pigment production, whereas **6** apparently affected pigment production, which was deemed different from that by **4a** especially in those iron-repleted conditions.

#### Catechol moiety of 4a is required for antipathogenic activity in *Drosophila*

**Figure 4.**
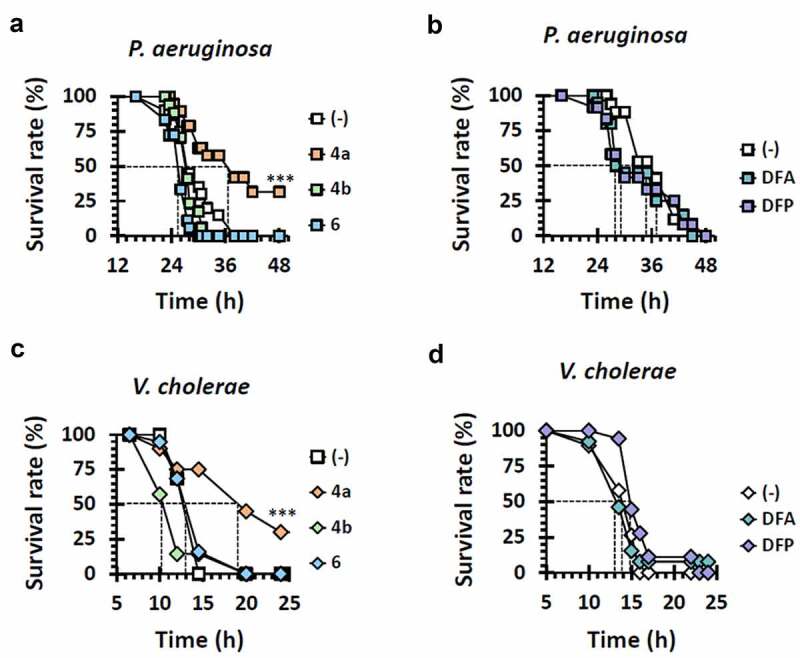
Antibacterial efficacy of other iron chelators. Mortality of infected flies upon administration of iron chelators were measured. The flies were systemically infected with *P. aeruginosa* PA14 (a and b) and *V. cholerae* N16961 (c and d). The infected flies were fed with either nothing (empty) or the designated chemicals as in Figure 3 and their survival rates were determined overtime. The dotted lines represent the time required to reach 50% mortality. The statistical significance based on a log-rank test is indicated (***, *p* < 0.001).

The data in Figure 3 prompted us to evaluate the antibacterial efficacy of those compounds in *Drosophila* infections. We first investigated whether those compounds could rescue the mortality of the PA14-infected flies, which were killed without antibacterial feeding within 50 h in our experimental condition. Figure 4a revealed the antibacterial activity of the **4a** analogs, where **4b** did not display antibacterial efficacy at all, suggesting that the catechol moiety of **4a** is crucial for its antibacterial efficacy. Since no growth inhibition was observed for PA14 even at the saturated concentration (~2 mM), the antibacterial activity of **4a** involving the catechol moiety is most likely due to its activity to chelate iron and thus to attenuate the virulence of PA14, substantiating that **4a** is a novel antipathogenic hit, although the mechanistic details into its mode of action need to be further investigated. We found that **6** was slightly toxic in *Drosophila* with any antibacterial activity examined, despite its iron-chelating activity (Figure 3). The two iron-chelating drugs (DFP and DFA) were also evaluated for the antibacterial efficacy, but they displayed no antibacterial activity in *Drosophila* infections (Figure 4b). It should be noted that DFP inhibited the growth of PA14 in vitro unlike **4a** (Figure 3). These results suggest that the antibacterial activity of **4a** is not attributed only to its iron-chelating property. Considering the data from **6**, the iron-chelating activity should be on the appropriate configuration for this antibacterial pharmacophore.

We also examined the chemicals for their in vivo antibacterial activity against *V. cholerae*. As shown in Figure 4c,d, the data paralleled those from the PA14 infections (Figure 4a,b). This result indicates that the similar mode of action might work for **4a** in *V. cholerae* as well, despite the lack of visible pigments like PYO and PVD in this bacterium.

#### Transcriptomic response to 4a is complex, compared with those to other chelators

**Figure 5.**
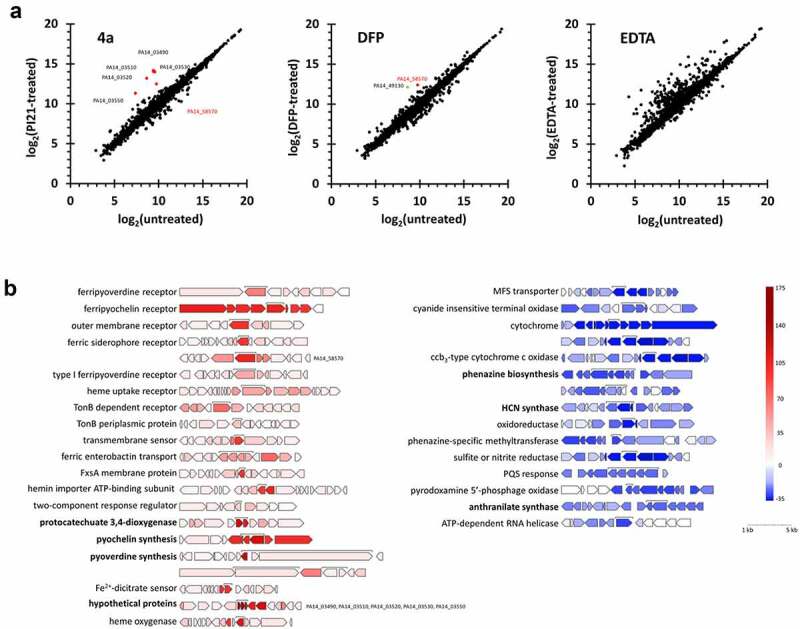
Transcriptome upon **4a**. (a). Scatter plots for the transcriptomes of the cells treated with **4a**, DFP, or EDTA. The x-axis represents the normalized data (log_2_) from the un-treated bacteria and the y-axis represents those from the bacteria treated with **4a**, DFP or EDTA at 200 µM. (b). Representative **4a**-responsive genes and operons in a. Genes with over 2-fold changes in the counted number per gene are marked as red (upregulated) and blue (downregulated) with the intensity scale at the right. The brackets indicate the highest or lowest expressed genes in a gene cluster with description. Each gene cluster is drawn to scale.

As an attempt to get an insight into the antipathogenic property of **4a** as a chelating agent, we carried out transcriptome profiling in response to the chelating agents. Figure 5a represents the transcriptomic snapshot from the PA14 cells that had been treated with **4a** in comparison with that from the cells treated with either DFP or EDTA (at 200 µM) due to the bactericidality of DFP and EDTA at 500 µM. EDTA treatment provoked the changes in relatively many genes, which might be attributed to its broad chelating spectrum [33].

**4a** and DFP at 200 µM showed similar gene expression profiles. Unlike DFP, however, **4a** significantly affected many genes (Figure 5a). The functions of representative differentially expressed genes (DEGs) are designated in Figure 5b, all of which are those upregulated by **4a**. For example, those for PVD and PCH synthesis and siderophore transporters such as FpvA and FpvB were significantly upregulated, clearly indicative of the iron starvation response upon iron depletion caused by **4a**. It is noteworthy that the *pcaGH* operon for protocatechuate 3,4-dioxygenase was highly upregulated (~40 folds), which is involved in protocatechuate cleavage for hydrocarbon degradation. It is presumable that the *pcaGH* upregulation is not directly associated with the iron starvation response, but with the catechol moiety of **4a**. Moreover, the three hypothetical genes in a row (PA14_03490, PA14_03510, and PA14_03520) were upregulated by over 100 folds, the function of which is yet to be discovered.

The down-regulated DEGs did not qualitatively differ between **4a** and DFP, despite some quantitative differences in fold changes, which needs to be further elucidated. The genes required for PYO biosynthesis was clearly down-regulated and those for known virulence factors such as hydrogen cyanide and the secreted toxin (ExoY) were down-regulated only by **4a**, which might account for the antipathogenic property of **4a**. The genes involved in respiratory electron transport and those for oxidoreductases (e.g. PA14_44260) belong to the highly down-regulated genes, which might be associated with the iron depletion caused by **4a**. Taken together, the transcriptomic response to **4a** should comprise the iron-sparing response, substantiating the function of **4a** in iron depletion, although it is still enigmatic that **4a**-mediated iron depletion would not lead to bacterial killing unlike DFP-mediated iron depletion, which will be further unveiled possibly by focusing on the unique DEGs upon **4a**.

## Discussion

In this study, inspired by a previous review article [15], we first exploited *Drosophila*-based non-mammalian model host infections to identify a new antibacterial hit, **4a**, which is a pyridine-3-*N*-sulfonylpiperidine, some related congeners of which have anti-metastatic activity in breast and melanoma cancer cell lines [26]. **4a** (the compound **3i** in the previous study) exhibited no cytotoxic activity and more importantly no toxicity in *Drosophila* feeding even at the saturated concentration (~2 mM) in the cornmeal media. Despite its structural similarity to the antifolates, sulfa drugs, **4a** did not inhibit the bacterial growth with its minimal inhibitory concentration undetermined. Although the chemical details regarding the bioactivity of **4a** are yet to be revealed, it became clear that the ability of **4a**-mediated attenuation of virulence is dependent on the iron-chelating activity of the 1,2-dihydroxybenzene (i.e. catechol) moiety, rather than other structural features. The structure–activity relationship study focusing on the sulfonamide substituents and/or the hydrazone linkers would help the unique bioactivity of **4a** in comparison with other iron-chelating drugs and congeners used in this study.

As a phenotype-based approach for a new class of anti-infectives, we hypothesized that a *Drosophila*-based screen could enable identification of small molecules that inhibit the virulence pathways of the bacteria or enhance the immune pathways of the *Drosophila*. In both cases, the identified hits could not directly inhibit the bacterial growth. Moy et al. [34] first identified antimicrobial compounds against *Enterococcus faecalis* using *C. elegans*-based live animal infection. They could classify the hit compounds as anti-infectives rather than traditional antibiotics because they rescued the infected animals without affecting the bacterial growth. They further validated this screen using live animal infection could reveal chemicals with novel modes of action and serve as a proof-of-concept study of complex biological processes in the interface between bacterial pathogens and host animals [14,34]. Despite the lack of automation and subsequently the difficulty of high throughputness as in *C. elegans* screen, our work is noteworthy, considering several advantages of *Drosophila* infections over *C. elegans* infections: *Drosophila* exhibits closer evolutionary relatedness to human than *C. elegans* in regards to innate immunity: for example, *C. elegans* lacks the canonical TLR signaling pathways for NF-κB activation, whereas *Drosophila* possess orthologous pathways (Toll and Imd) to activate Dorsal, Dif and Relish [35]. More microbial infection models have been established for *Drosophila* than for *C. elegans* [36]. More importantly, polymicrobial infection models could be more readily established for *Drosophila* based on the complicated organ and microbiome structures [19,37,38].

The most interesting and puzzling observation in this work is that **4a**-mediated iron depletion would not result in bacterial killing. This is quite unique to this compound in that DFP and EDTA are bactericidal. It is also understandable that DFA did not affect the growth at all, since it is a well-known xenosiderophore [32]. This may lead us to a simple hypothesis that **4a** would act as a xenosiderophore as well. Siderophores that chelates insoluble iron (III) become imperative for many bacterial pathogens in the host environments and ablation of this system significantly attenuates the pathogenesis [39,40]. It is possible that **4a** could attenuate virulence at certain concentrations, although it would indeed function as a xenosiderophore at other concentrations for *P. aeruginosa*. Alternatively, the impact of **4a**-mediated iron chelation might lie in perturbing the iron homeostasis during bacterial growth and survival, based on its chelating activity toward both iron (II) and iron (III) at similar efficiency. Although iron is an essential element for the growth and survival for most microorganisms, high levels of free iron (III) may result in progressive cell damage via the Fenton reaction that generates hydroxyl radicals under certain circumstances. Thus, due to the nature of iron as a two-edged sword, the effect of iron (II) and (III) chelation by **4a** needs to be more quantitatively investigated both in vitro and in vivo to understand the antipathogenic property of **4a**. The comparison between the transcriptomic responses to **4a** and EDTA, which does not chelate iron (III), might be helpful to understand the unique feature of **4a**. Moreover, the examination of the synthetic congeneric compound, **6** (containing hydroxamate rather than catechol) which chelates iron (II) and iron (III) at different efficiency, would enable us to delve into the mechanistic details of **4a** effect.

## Supplementary Material

Supplemental Material

## Data Availability

The data that support the findings of this study are available from the corresponding author upon reasonable request.

## References

[1] Prestinaci F, Pezzotti P, Pantosti A. Antimicrobial resistance: a global multifaceted phenomenon. Pathog Glob Health. 2015;109(7):309–318. 26343252 10.1179/2047773215Y.0000000030 PMC4768623

[2] Cabot G, Zamorano L, Moyà B, et al. Evolution of *Pseudomonas aeruginosa* antimicrobial resistance and fitness under low and high mutation rates. Antimicrob Agents Chemother. 2016;60(3):1767–1778. DOI:10.1128/AAC.02676-15 26729493 PMC4775977

[3] Mulani MS, Kamble EE, Kumkar SN, et al. Emerging strategies to combat ESKAPE pathogens in the era of antimicrobial resistance: a review. Front Microbiol. 2019;10:539. 30988669 10.3389/fmicb.2019.00539 PMC6452778

[4] Kirienko DR, Kang D, Kirienko NV. Novel pyoverdine inhibitors mitigate *Pseudomonas aeruginosa* pathogenesis. Front Microbiol. 2019;9:3317. 30687293 10.3389/fmicb.2018.03317 PMC6333909

[5] Rasko DA, Sperandio V. Anti-Virulence strategies to combat bacteria-mediated disease. Nat Rev Drug Discov. 2010;9(2):117–128. 20081869 10.1038/nrd3013

[6] Ofek I, Hasty DL, Doyle RJ. Bacterial adhesion to animal cells and tissues. Vol. 1725. Washington, DC: ASM. press;2003. DOI:10.1128/9781555817800

[7] Deep A, Chaudhary U, Gupta V. Quorum sensing and bacterial pathogenicity: from molecules to disease. J Lab Physicians. 2011;3(01):004–011. 10.4103/0974-2727.78553 PMC3118056 21701655

[8] Rutherford ST, Bassler BL. Bacterial quorum sensing: its role in virulence and possibilities for its control. Cold Spring Harb Perspect Med. 2012;2(11):a012427. 23125205 10.1101/cshperspect.a012427 PMC3543102

[9] Oh HY, Jalde SS, Chung IY, et al. An antipathogenic compound that targets the OxyR peroxide sensor in *Pseudomonas aeruginosa*. J Med Microbiol. 2021;70(4):001341. DOI:10.1099/jmm.0.001341 PMC8289212 33830911

[10] Chung IY, Jang HJ, Bae HW, et al. A phage protein that inhibits the bacterial ATPase required for type IV pilus assembly. Proc Natl Acad Sci USA. 2014;111(31):11503–11508. DOI:10.1073/pnas.1403537111 25049409 PMC4128137

[11] Bo K, Jang HJ, Chung IY, et al. Nitrate respiration promotes polymyxin B resistance in *Pseudomonas aeruginosa*. Antioxid Redox Signal. 2021;34(6):442–451. DOI:10.1089/ars.2019.7924 32370551

[12] O’-Callaghan D, Vergunst A. Non-Mammalian animal models to study infectious disease: worms or fly fishing? Curr Opin Microbiol. 2010;13(1):79–85. 20045373 10.1016/j.mib.2009.12.005

[13] Lorenz A, Pawar V, Häussler S, et al. Insights into host–pathogen interactions from state‐of‐the‐art animal models of respiratory *Pseudomonas aeruginosa* infections. FEBS Lett. 2016;590(21):3941–3959. DOI:10.1002/1873-3468.12454 27730639

[14] Moy TI, Conery AL, Larkins-Ford J, et al. High-Throughput screen for novel antimicrobials using a whole animal infection model. ACS Chem Biol. 2009;4(7):527–533. DOI:10.1021/cb900084v 19572548 PMC2745594

[15] Tzelepis I, Kapsetaki SE, Panayidou S, et al. *Drosophila melanogaster*: a first step and a stepping-stone to anti-infectives. Curr Opin Pharmacol. 2013;13(5):763–768. DOI:10.1016/j.coph.2013.08.003 23992884 PMC7185596

[16] Pukkila-Worley R, Feinbaum R, Kirienko NV, et al. Stimulation of host immune defenses by a small molecule protects *C. elegans* from bacterial infection. PLoS Genet. 2012;8(6):e1002733. DOI:10.1371/journal.pgen.1002733 22719261 PMC3375230

[17] Dach K, Yaghoobi B, Schmuck MR, et al. Teratological and behavioral screening of the national toxicology program 91-compound library in zebrafish (*Danio rerio*). Toxicol Sci. 2019;167(1):77–91. DOI:10.1093/toxsci/kfy266 30364989 PMC6317431

[18] Jang HJ, Chung IY, Lim C, et al. Redirecting an anticancer to an antibacterial hit against methicillin-resistant *Staphylococcus aureus*. Front Microbiol. 2019;10:350. 30858845 10.3389/fmicb.2019.00350 PMC6398426

[19] Lee YJ, Jang HJ, Chung IY, et al. *Drosophila melanogaster* as a polymicrobial infection model for *Pseudomonas aeruginosa* and *Staphylococcus aureus*. J Microbiol. 2018;56(8):534–541. DOI:10.1007/s12275-018-8331-9 30047081

[20] Kim SH, Park SY, Heo YJ, et al. *Drosophila melanogaster*-based screening for multihost virulence factors of *Pseudomonas aeruginosa* PA14 and identification of a virulence-attenuating factor, HudA. Infect Immun. 2008;76(9):4152–4162. DOI:10.1128/IAI.01637-07 18591226 PMC2519450

[21] Burke RM, Upton ME, McLoughlin AJ. Influence of pigment production on resistance to ultraviolet irradiation in *Pseudomonas aeruginosa* ATCC 10145. Irish J Food Sci Technol. 1990;14(1):51–60.

[22] Meyer JM, Abdallah MA. The fluorescent pigment of *Pseudomonas fluorescens*: biosynthesis, purification and physicochemical properties. Microbiology. 1978;107(2):319–328.

[23] Imperi F, Tiburzi F, Visca P. Molecular basis of pyoverdine siderophore recycling in *Pseudomonas aeruginosa*. Proc Natl Acad Sci, USA. 2009;106(48):20440–20445. 19906986 10.1073/pnas.0908760106 PMC2787144

[24] Gentleman RC, Carey VJ, Bates DM, et al. Bioconductor: open software development for computational biology and bioinformatics. Genome Biol. 2004;5(10):1–16. DOI:10.1186/gb-2004-5-10-r80 PMC545600 15461798

[25] Chan SWL, Henderson IR, Jacobsen SE. Gardening the genome: DNA methylation in *Arabidopsis thaliana*. Nat Rev Genet. 2005;6(5):351–360. 15861207 10.1038/nrg1601

[26] Kang SM, Nam KY, Jung SY, et al. Inhibition of cancer cell invasion by new ((3, 4-dihydroxy benzylidene) hydrazinyl) pyridine-3-sulfonamide analogs. Bioorg Med Chem Lett. 2016;26(4):1322–1328. DOI:10.1016/j.bmcl.2015.12.093 26810259

[27] Zhang Q, Jin B, Wang X, et al. The mono (catecholamine) derivatives as iron chelators: synthesis, solution thermodynamic stability and antioxidant properties research. R Soc Open Sci. 2018;5(6):171492. DOI:10.1098/rsos.171492 30110407 PMC6030290

[28] Wang Y, Newman DK. Redox reactions of phenazine antibiotics with ferric (hydr)oxides and molecular oxygen. Environ Sci Technol. 2008;42(7):2380–2386. 18504969 10.1021/es702290a PMC2778262

[29] Pierson LS, Pierson EA. Metabolism and function of phenazines in bacteria: impacts on the behavior of bacteria in the environment and biotechnological processes. Appl Microbiol Biotechnol. 2010;86(6):1659–1670. 20352425 10.1007/s00253-010-2509-3 PMC2858273

[30] Rodríguez-Rojas A, Makarova O, Müller U, et al. Cationic peptides facilitate iron-induced mutagenesis in bacteria. PLoS Genet. 2015;11(10):e1005546. DOI:10.1371/journal.pgen.1005546 26430769 PMC4592263

[31] Wang R, An L, He J, et al. A class of water-soluble Fe (III) coordination complexes as *T*_1_-weighted MRI contrast agents. J Mater Chem B. 2021;9(7):1787–1791. DOI:10.1039/d0tb02716b 33595044

[32] Symeonidis AS. The role of iron and iron chelators in zygomycosis. Clin Microbiol Infect. 2009;15:26–32. 10.1111/j.1469-0691.2009.02976.x 19754753

[33] Kroeger T, Frieg B, Zhang T, et al. EDTA aggregates induce SYPRO orange-based fluorescence in thermal shift assay. PLoS One. 2017;12(5):e0177024. DOI:10.1371/journal.pone.0177024 28472107 PMC5417642

[34] Moy TI, Ball AR, Anklesaria Z, et al. Identification of novel antimicrobials using a live-animal infection model. Proc Natl Acad Sci, USA. 2006;103(27):10414–10419. DOI:10.1073/pnas.0604055103 16801562 PMC1482800

[35] Hoffmann JA, Reichhart JM. *Drosophila* innate immunity: an evolutionary perspective. Nat Immunol. 2002;3(2):121–126. 11812988 10.1038/ni0202-121

[36] Glavis-Bloom J, Muhammed M, Mylonakis E. Of model hosts and man: using *Caenorhabditis elegans*, *Drosophila melanogaster* and *Galleria mellonella* as model hosts for infectious disease research. Adv Exp Med Biol. 2012;11–17. DOI:10.1007/978-1-4419-5638-5_2 22127881

[37] Sibley CD, Duan K, Fischer C, et al. Discerning the complexity of community interactions using a *Drosophila* model of polymicrobial infections. PLoS Pathog. 2008;4(10):e1000184. DOI:10.1371/journal.ppat.1000184 18949036 PMC2566602

[38] Su CY, Menuz K, Carlson JR. Olfactory perception: receptors, cells, and circuits. Cell. 2009;139(1):45–59. 19804753 10.1016/j.cell.2009.09.015 PMC2765334

[39] Bakkeren E, Diard M, Hardt WD. Evolutionary causes and consequences of bacterial antibiotic persistence. Nat Rev Microbiol. 2020;18(9):479–490. 32461608 10.1038/s41579-020-0378-z

[40] Ruff WE, Greiling TM, Kriegel MA. Host–microbiota interactions in immune-mediated diseases. Nat Rev Microbiol. 2020;18(9):521–538. 32457482 10.1038/s41579-020-0367-2

