# Supplementary material for: An iron-chelating sulfonamide identified from Drosophila-based screening for antipathogenic discovery
Source: Virulence. 2022 May 6;13(1):833–43. doi: 10.1080/21505594.2022.2069325 (PMC9090290; doi:10.1080/21505594.2022.2069325)
Supplement: Supplemental Material [file KVIR_A_2069325_SM0404.zip › supplementary/YooYJ_PI21_Virulence_SI_R1_clean.docx]

# Supplemental Material

An iron-chelating sulfonamide identified from *Drosophila*-based screening for antipathogenic discovery

Yeon-Ji Yoo,^1^ In-Young Chung,^1^ Shivakumar S. Jalde,^2^ Hyun-Kyung Choi,^2^* and You-Hee Cho^1^*

*^1^Department of Pharmacy, College of Pharmacy and Institute of Pharmaceutical Sciences, CHA University, Gyeonggi-do 13488, Korea and ^2^Department of Chemistry, Sogang University, 35 Baekbeom-ro, Mapo-gu, Seoul 04107, Korea*

^*^ To whom correspondence should be addressed. Tel: +82-31-881-7165; Fax: +82- 31-881-7219; E-mail: [youhee@cha.ac.kr](mailto:youhee@cha.ac.kr)

Correspondence may also be addressed to Hyun-Kyung Choi. Tel: +82-2-715-0797; Fax: +82-2-718-4218; E-mail: [hkchoi45@sogang.ac.kr](mailto:hkchoi45@sogang.ac.kr)

Running Title: Antipathogenic iron chelating sulfonamide from *Drosophila* screening

Keywords: iron, chelator, catechol, sulfonamide, antipathogenic, virulence, *Drosophila*, *Pseudomonas aeruginosa*

# Supplemental Methods

## Synthesis of the iron-chelating 4a derivatives

**(7a)** To a solution of **4a** (50 mg, 0.13 mmol, 1 eq) in ACN (10 ml) was added FeCl_3_.6H_2_O (72 mg, 0.26 mmol, 2 eq) in water (1 ml) and then stirred at RT for 12 h. The reaction mixture was evaporated under vacuo, diluted with 20% MeOH in MC and washed and concentrated to afford 46 mg (29.30%) of the desired compound. IR (ν_max_) [1]: 1654, 1604, 1341, 1283, 1170, 1107, 592 cm^−1^, UV: The absorbance peak was found at 486 nm.

**(7b)** To a solution of **4a** (50 mg, 0.13 mmol, 1 eq) in ACN (10 ml) was added FeSO_4_.7H_2_O (72 mg, 0.26 mmol, 2 eq) in water (1 ml) and then stirred at RT for 12 h. The reaction mixture was prepared as for **7a** to afford 41 mg (38.31%) of the desired compound. IR (ν_max_) [1]: 1590, 1281, 1166, 1135, 1107, 730, 591 cm^−1^, UV: The absorbance peak was found at 486 nm.

# Supplemental Figures and tables


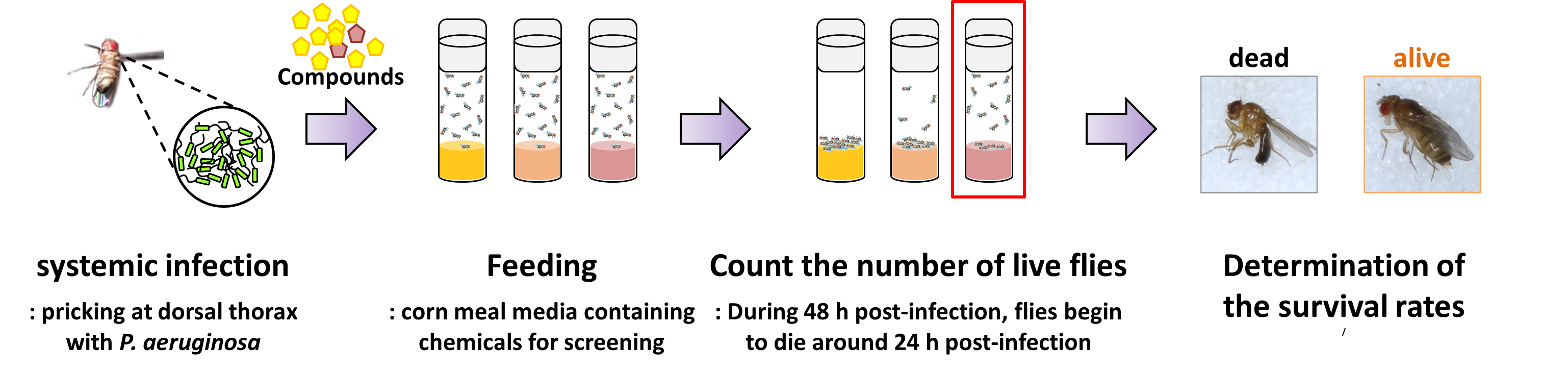


## Figure S1. Schematic representation of the *Drosophila*-based screening

The whole procedure consists of the following steps: the infected flies were transferred to the medium that had been overlaid with the compounds. The compounds with survival rate of 33% or more at 50-h post-infection were selected as primary hits. After repeated experiments, three compounds were selected as the final hits from 444 in-house chemicals and drugs. The details of the *Drosophila* experiments are described in Materials and Methods.


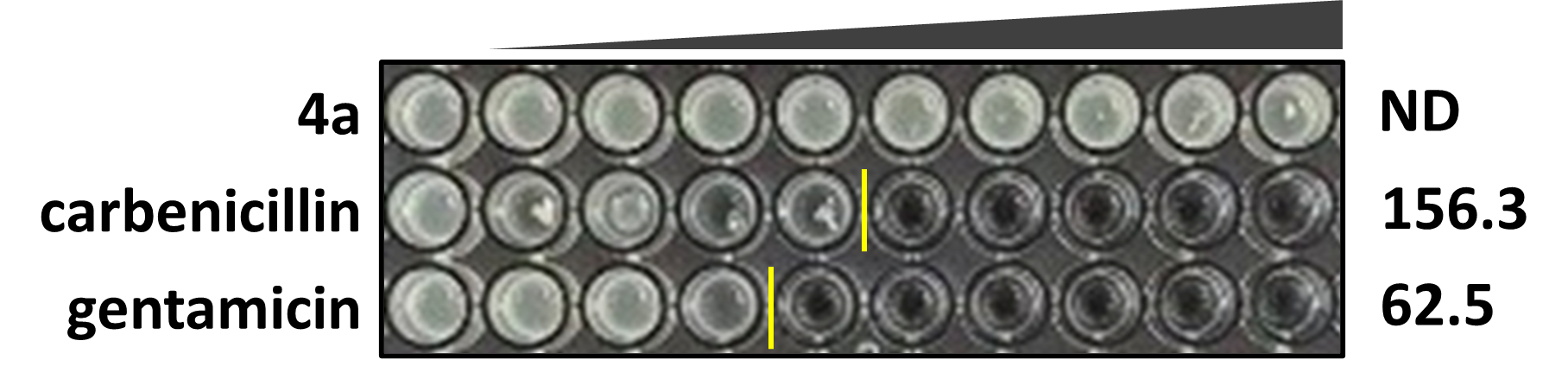


## Figure S2. Minimal inhibitory concentration of 4a.

The MIC of **4a** was measured using *P. aeruginosa* PA14, alongside carbenicillin and gentamicin as the controls. The numbers at the right indicate the MICs (μM), with the vertical yellow lines denoting the boundaries between MICs and the maximal growth-permitting concentrations. ND indicates not determined at the concentrations up to 1 mM.


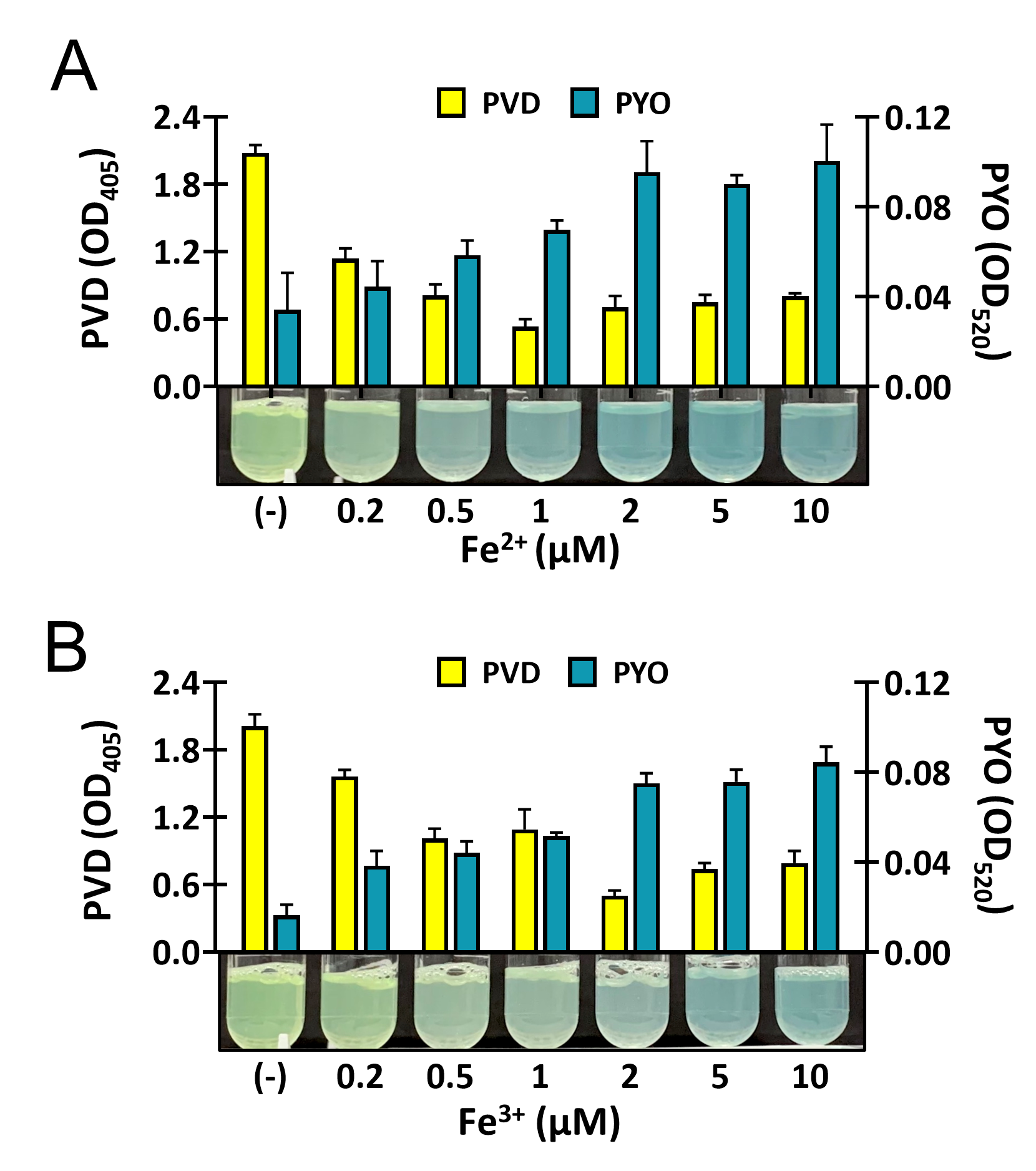


## Figure S3. Iron repletion into minimal media

The bacterial cells were grown to the late stationary phase in M9-citrate minimal media supplemented with the indicated concentrations of FeSO_4_ (Fe^2+^, A) and FeCl_3_ (Fe^3+^, B). The amounts of extracellular pyoverdine (PVD) (yellow, left y axis) and pyocyanin (PYO) (green, right y axis) were measured as OD_405_ and OD_520_ respectively, using the PA14 cell-free culture supernatants. Pigment production of the cultures by visual inspection was shown at the bottom of the graphs.


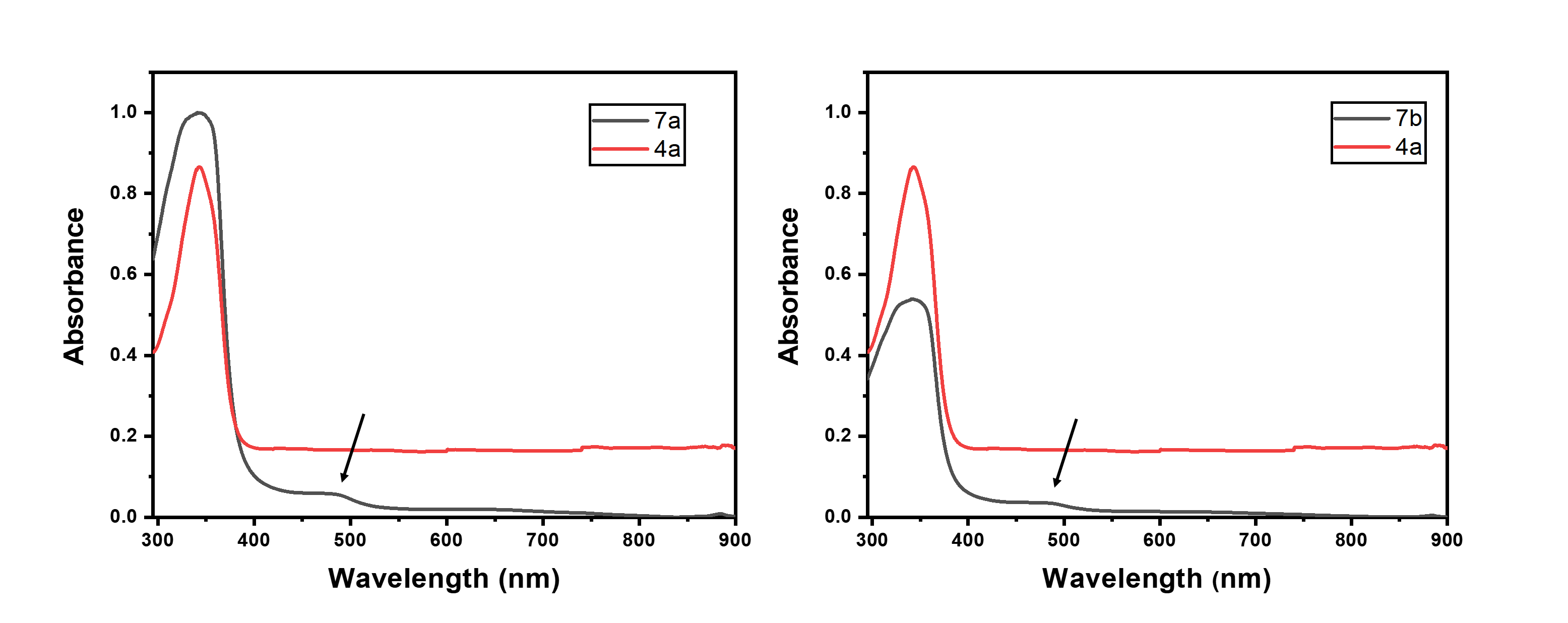


## Figure S4. UV-Vis spectra of the 4a-iron complexes

UV-Vis spectra of the **4a** compounds chelated with Fe^3+^ (**7a**) and Fe^2+^ (**7b**) in 20% MeOH + DCM. The absorptions peak near 486 nm in the UV-vis absorption spectra of the compounds **7a** and **7b** were allocated to the d–d electronic transition as indicated by arrows.

## Figure S5. Chemical structures of the pyridine-3-*N*-sulfonylpiperidine derivatives

The identified antipathogenic hit (**4a**) and its derivatives, **4b** and **6**, are shown: **4b** has 1,2-dimethoxy substituents at the **4a** catechol moiety; **6** has hydroxamate instead of the 1,2-diol of **4a**.

# Supplemental References

1. Wang R, An L, He J, et al. A class of water-soluble Fe (III) coordination complexes as *T*_1_-weighted MRI contrast agents. J. Mater. Chem. B. 2021;9(7): 1787-1791. DOI: 10.1039/d0tb02716b.
