# Supplementary figures and images for: An iron-chelating sulfonamide identified from Drosophila-based screening for antipathogenic discovery
Source: Virulence. 2022 May 6;13(1):833–43. doi: 10.1080/21505594.2022.2069325 (PMC9090290; doi:10.1080/21505594.2022.2069325)

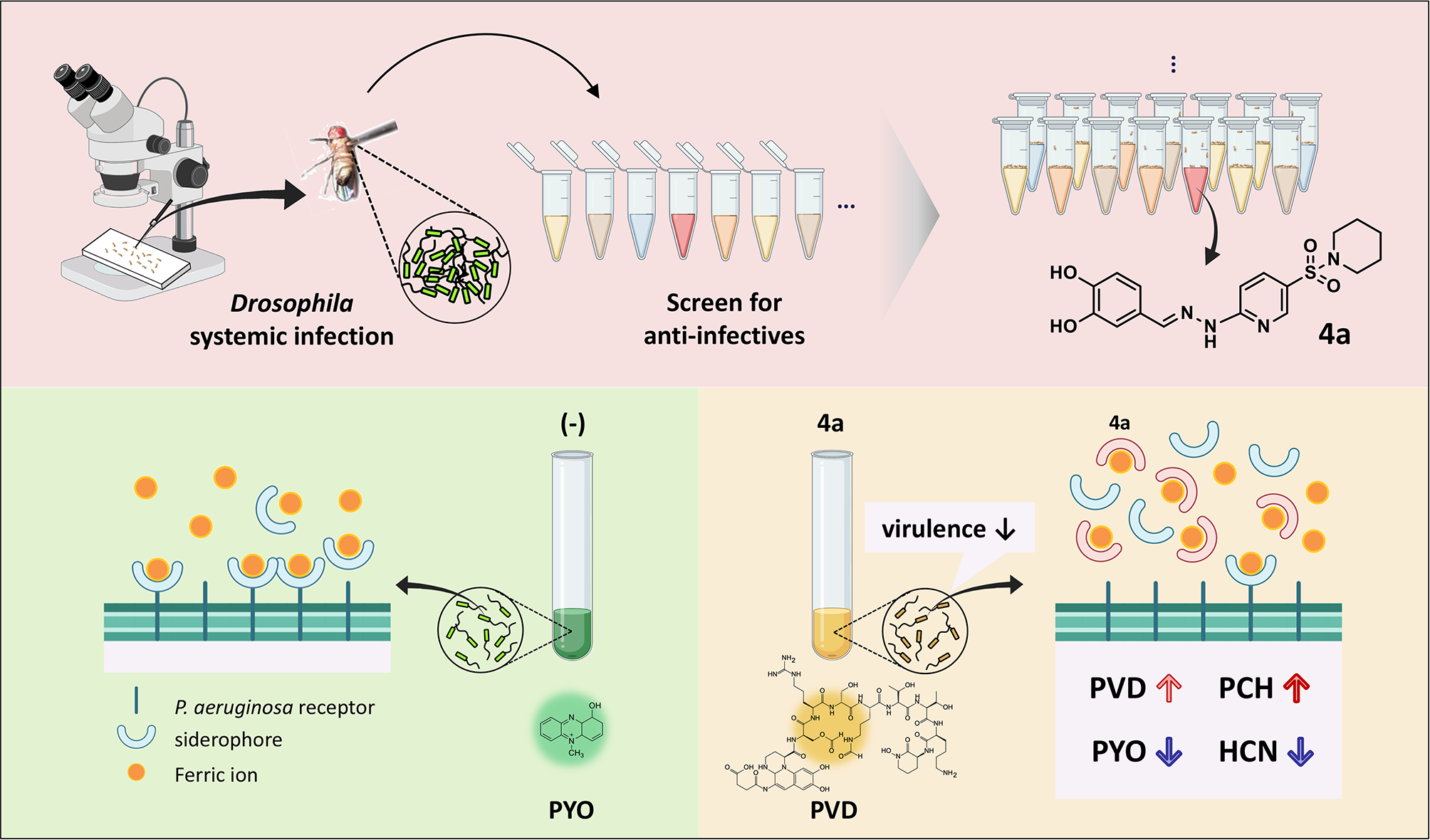

Supplement: Supplemental Material [file KVIR_A_2069325_SM0404.zip › supplementary/YooYJ_PI21_Graphical_R1.tif]
